# Obstetrics and gynecology residents’ satisfaction and self-confidence after an anal sphincter injury simulation-based workshop in Indonesia: a pre- and post-intervention comparison study

**DOI:** 10.3352/jeehp.2022.19.4

**Published:** 2022-02-14

**Authors:** Riska Wahyuningtyas, Eighty Mardiyan Kurniawati, Budi Utomo, Gatut Hardianto, Hari Paraton, Tri Hastono, Djoko Kuswanto

**Affiliations:** 1 Obstetrics and Gynecology Department, Faculty of Medicine, Universitas Airlangga, Surabaya, Indonesia; 2 Urogynecology Reconstruction Division, Obstetrics and Gynecology Department, Faculty of Medicine, Universitas Airlangga, Surabaya, Indonesia; 3 Public Health-Preventive Medicine, Faculty of Medicine, Universitas Airlangga, Surabaya, Indonesia; 4 Integrated Digital Design Laboratory, Design Product Department, Institut Teknologi Sepuluh Nopember, Surabaya, Indonesia; Hallym University, Korea

**Keywords:** Anal canal, Manikins, Obstetric delivery, Personal satisfaction, Silicones

## Abstract

**Purpose:**

Obstetric anal sphincter injury is one of the most common complications during delivery. Simulation models with manikins can be used as an effective medical learning method to improve students’ abilities before encountering patients. The present study aimed to describe the development of an anal sphincter injury model and to assess residents’ satisfaction and self-confidence after a perineal repair workshop with an anal sphincter injury simulator in Indonesia.

**Methods:**

This was a cross-sectional study with evaluation of outcomes before and after the workshop. We created a silicone-latex simulation anal sphincter injury model. Then, we validated this simulation and used it as a simulation model for the workshop. We asked residents about their satisfaction with repairing anal sphincter injuries using a simulation model and residents’ self-confidence when practicing anal sphincter injury repair.

**Results:**

All residents felt the simulation-based workshop was valuable (100%). Most of the scores for the similarity of the simulation model were good (about 8 out of maximum 10). The self-assessment of confidence was measured before and after the workshop. Overall self-confidence increased significantly after the workshop in identifying the external sphincter ani (EAS) (P=0.031), suturing the anal mucosa (P=0.001), suturing the internal sphincter ani (P=0.001), suturing the EAS (P<0.001), and evaluating the sphincter ani tone (P=0.016).

**Conclusion:**

The anal sphincter injury simulator improved residents’ self-confidence in identifying the EAS, suturing the anal mucosa, suturing the internal sphincter ani, suturing the EAS, and evaluating sphincter ani tone.

## Introduction

### Background/rationale

Obstetric anal sphincter injury, a 3rd- and 4th-degree injury, is one of the most common complications during delivery. It is clinically diagnosed in 11% of women who deliver vaginally [1]. The most common long-term problems of obstetric anal sphincter injury are dyspareunia, perineal pain, and flatus and fecal incontinence [2].

A study from the United States found that 60% of obstetrics and gynecology residents who underwent an education program did not have training in perineal rupture repair. Perineal repair is one of the obligatory competencies of residents, including obstetric anal sphincter injury repair [3]. Simulation models with manikins can be used as an effective medical learning method to improve students’ abilities before encountering patients. Thus, using simulators with repetitions can be a good way of learning skills and does not harm the patient.

The study of Banks et al. [4] on residents who were given perineal rupture repair training with a simulation model showed an increase in knowledge and skills, especially in first-level residents. Other studies have developed simulation models with materials from animal limbs such as goat perineum [5], cow tongue [6,7], and pig tongue and intestines [8]. However, the use of animals as the material of simulators can disrupt the ecosystem, provoke ethical problems, and is not durable. Furthermore, the anatomical structure is not similar to that of the human perineum; in particular, the goat’s perineum has a sphincter muscle that is not the same as the human perineum with a thin perineal body [5]. Currently, simulation models with silicone materials with detailed anatomical structures are marketed abroad at high prices.

### Objectives

The purpose of this study is to describe the development of a new silicone-latex anal sphincter injury model, which can be a pilot model for understanding the anatomical details of the perineum. This study also presents an assessment of residents’ satisfaction and self-confidence before and after the perineal repair workshop with the silicone-latex anal sphincter injury simulator.

## Methods

### Ethics statement

This study was approved by the ethics committee of General Soetomo Hospital, Surabaya (no., 0269/KEPK/IX/2021). Informed consent was obtained from all participants.

### Study design

This was a cross-sectional observational study with evaluation of outcomes before and after the intervention. The description was based on the STROBE (Strengthening the Reporting of Observational Studies in Epidemiology) statement.

### Setting

This study was conducted for 3rd- and 4th-year obstetrics and gynecology residents at Universitas Airlangga from July to November 2021. They performed 4th-degree laceration repair (anal sphincter injury repair) in the silicone-latex anal sphincter model before taking part in the workshop (pretest) then they did it again 1 week after the workshop session (posttest). Survey questionnaires were provided to residents before and after the workshop.

### Participants

All 22 residents who agreed to complete the study were included in this study. Residents who had no previous experience in repairing 3rd- and 4th-degree perineal laceration independently in patients were included in the study. There were no exclusion criteria.

### Variables

There were 2 variables measured in this study: residents’ satisfaction and self-confidence before and after the simulation workshop. All data were collected before and after the simulation workshop from the participants by questionnaire. Residents’ satisfaction was measured using a questionnaire about the evaluation of the model simulation. The authors developed the questionnaire after validation testing and included questions on the similarity of the model simulation to native perineal tissue, the flexibility of simulator tissue, and the similarity of the anal mucosa, internal sphincter ani (IAS), external sphincter ani (EAS), transversus perineal muscle, bulbocavernosus muscle, vaginal mucosa, and perineal skin to the native perineal tissue. This questionnaire consisted of a 10-point Likert scale, ranging from strongly unsatisfied to strongly satisfied, with a higher score indicating higher satisfaction levels (Supplement 1).

Residents’ self-confidence when practicing the anal sphincter injury repair was assessed. This variable was measured using a questionnaire consisting of “yes” and “no” questions on how confidently residents did each repair procedure, such as identifying the grade of laceration, anal mucosa, EAS and IAS, suturing the anal mucosa, EAS and IAS, vaginal mucosa, perineal muscle, perineal skin, evaluation sphincter ani tone, identifying the apex of vaginal mucosa or hymen, handling instruments, and selecting an appropriate needle and suture. This questionnaire was validated by the authors. The Cronbach’s α values of residents’ satisfaction and self-confidence were 0.76 and 0.90, respectively.

### Data source: simulation workshop

Leading up to the simulation session, residents were given an online seminar and video tutorial on 4th-degree perineal laceration (Supplement 2). Residents then practiced their repair skills using the silicone-latex anal sphincter injury simulator supervised by a urogynecologist. The model of the simulation consisted of a base and 1 replaceable perineal tear that could be used 8 times before replacement.

### Simulator development

We created a model of anal sphincter injury using Fusion360 (Autodesk Inc., San Rafael, CA, USA); then, we discussed the details of the models based on an anatomy book. The study was conducted in the Integrated Digital Design Laboratory, Design Product Department, Institut Teknologi Sepuluh Nopember, from September to October 2021.

**Fig. 1.**
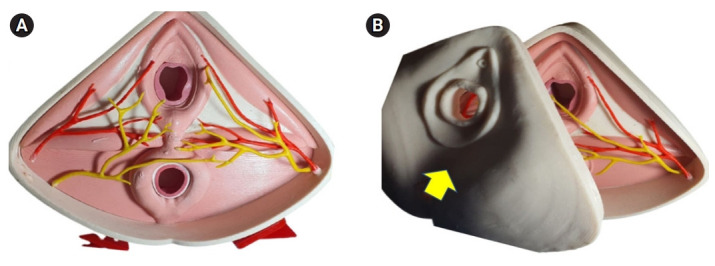
Perineal model. (A) Perineal model for learning anatomy. (B) Perineal model covered by perineal skin (yellow arrow).

**Fig. 2.**
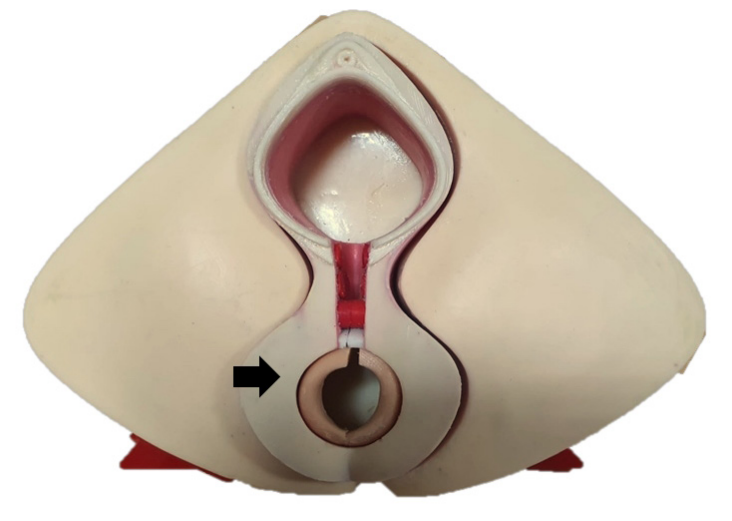
Anal sphincter injury model with a replaceable perineal pad (black arrow).

**Fig. 3.**
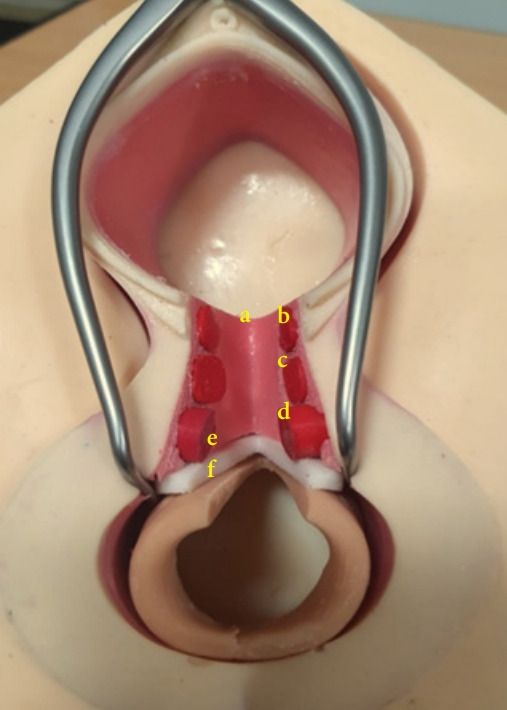
Detailed anatomy of the anal sphincter injury model. a, vaginal lumen; b, bulbocavernosus muscle; c, transversus perineal muscle; d, external an al sphincter; e, internal anal sphincter; f, anal mucosa.

We produced 2 types of simulators: first, the perineal model with nerve and blood supply of the pudendum that can be a model for learning perineal anatomy, and second, an anal sphincter injury model that showed 4th-degree perineal laceration. The residents repaired 4th-degree perineal laceration using the new silicone-latex anal sphincter injury simulator (Figs. 1–3).

### Study size

All obstetrics and gynecology residents who met the inclusion criteria and agreed to join this study were included. There was no estimation of sample size.

### Bias

There was a low risk of selection and performance bias. We used a specially developed and validated instrument for data collection to eliminate measurement bias.

### Statistical methods

The McNemar test was used to evaluate the difference in residents’ self-confidence before and after the simulation using IBM SPSS ver. 26.0 (IBM Corp., Armonk, NY, USA). A P-value less than 0.05 was considered statistically significant.

## Results

### Participants and descriptive data

Twenty-two obstetrics and gynecology residents from Universitas Airlangga, Surabaya/Soetomo General Hospital attended the workshop and responded to the survey with 100% response rate. The mean age of residents was 31 (±2.8) years , 54.5% were male residents, and 45.5% were female residents. The distribution of residents according to year in residency was equal between 3rd-year residents (50%) and 4th-year residents (50%). Most residents (77.3%) had assisted in anal sphincter injury repair fewer than 3 times. Most residents received their knowledge of anal sphincter injury repair from the lecture (50%) (Table 1). All residents felt that the simulation-based workshop was valuable (100%) and they hoped this training would be applied before they performed the repair in the patients. Residents’ satisfaction with the model simulation is presented in Table 2. Most of the scores for the similarity simulation model were good (about 8).

### Main results

The self-assessment of confidence was measured prior to the workshop and after the workshop. The overall self-confidence increased significantly after the workshop in identifying the EAS (P=0.031), suturing the anal mucosa (P=0.001), suturing the IAS (P=0.001), suturing the EAS (P<0.001), and evaluating the sphincter ani tone (P=0.016) (Table 3, Dataset 1).

**Fig. 4.**
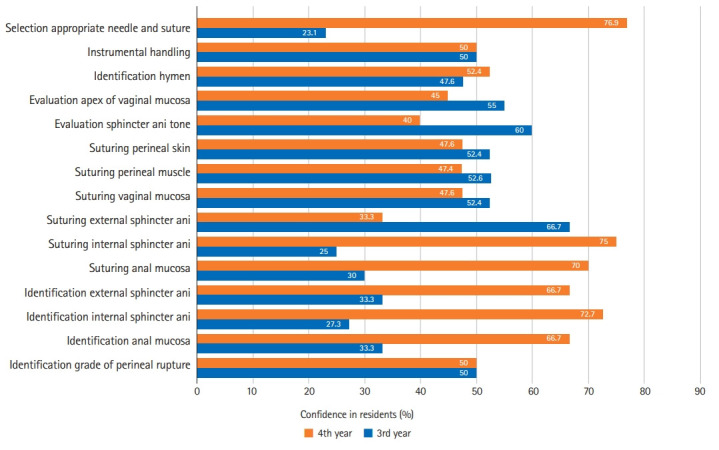
Percentage of confidence in residents based on year in residency before simulation-based workshop.

Self-confidence before the workshop was evaluated based on the year in residency. There were significant differences between 3rd- and 4th-year residents in identifying the anal mucosa (P=0.045), identifying the IAS (P=0.043), identifying the EAS (P=0.032), and selecting an appropriate needle and suture (P=0.004) (Fig. 4, Dataset 2).

**Fig. 5.**
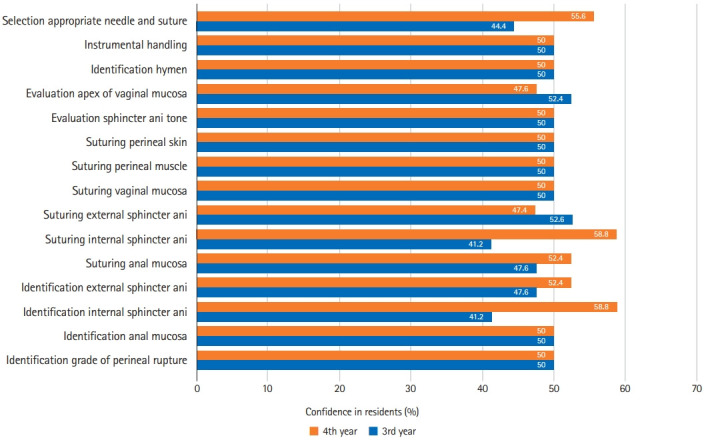
Percentage of confidence in residents based on year in residency after simulation-based workshop.

Self-confidence after the workshop was also evaluated based on the year in residency. There was no significant difference between the 3rd- and 4th-year residents in all procedures. In other words, after the simulation-based workshop the self-confidence of residents was the same (Fig. 5).

## Discussion

### Key results

The overall self-confidence of obstetrics and gynecology residents in identification procedures increased significantly after the anal sphincter injury simulation-based workshop.

### Interpretation

This study is the first observational study of the creation of a perineal simulator in Indonesia using a 3-dimensional (3D)-printed mold. Goudie et al. [9] also developed an anatomical silicone model of anal sphincter injury using a 3D-printed mold, and they found it a cost-effective model of obstetric anal sphincter injury repair and an effectively understandable learning tool for midwives, residents, and doctors.

This innovative model is a well-received and affordable teaching tool with excellent materials. As simulation becomes increasingly important in medical education, particularly for 3rd- and 4th-degree laceration repair, this model could be a valuable adjunctive curricular component. Several studies have evaluated models for teaching obstetric anal sphincter injury repair and have found improvements in resident skill sets after undergoing an educational workshop [10-12].

The perineal latex-silicone model was thought to be superior as compared to an animal model or sponge model, since it provided an anatomically correct simulation tool for learning to repair the anal sphincter. The models were also very durable in that they showed very few signs of wear or tear after attempts by 22 residents from our institution; each replaceable pad can be used by 8 participants. We added vessels and nerves to this model with details related to the anal sphincter for learning anatomy. Thus, we considered this simulation model to be a high-fidelity model for teaching perineal laceration repair.

Residents reported high acceptance and satisfaction with this anal sphincter injury model. Davis et al. [13] explained that self-assessment has limitations, as with easy tasks, participants tend to overestimate their abilities or skills, whereas with difficult tasks, good performers tend to underestimate to a lesser extent. However, asking about confidence is important to know whether a workshop has an effect on a specific technical skill [13]. No previous study has published a self-confidence assessment for anal sphincter injury repair, so this is the first study to establish self-confidence in residents before and after a simulation-based workshop.

### Comparison with previous studies

Andrews et al. [14] found that there were significant improvements in overlap repair, selecting an appropriate suture material for repairing the EAS, identifying the IAS, and repairing the IAS after a simulation-based-workshop. Thus, in our native model, identification and repairing the EAS and IAS were difficult due to the same gross anatomy. Using imaging modalities can increase the incidence of EAS tearing to 11%, and with multiple examiners, obstetric anal sphincter injuries identification increased to 25%. The low confidence could be caused by a low rate of training, although 50% of residents perform anal sphincter injury repair at some time. In the study of McLennan et al. [15], 60% of trainees had not received didactic training on techniques for perineal repair, and 50% of trainees had not received formal teaching on pelvic floor anatomy. Only 3% of residents in this study underwent a workshop session in another program. Furthermore, the frequency of anal sphincter repair in most residents was fewer than 3 times, so the residents had a low rate of training, and were exposed to the technique of repair.

We found no significant difference in each technique of anal sphincter repair between 3rd- and 4th-year residents after a simulation-based workshop. However, in the early test before the simulation-based workshop we found significantly different self-confidence selecting an appropriate needle and suture material, identifying the anal mucosa, identifying the IAS, and identifying the EAS. This study is comparable to that of Banks et al. [4], in which significant improvements in repair performance were seen in first-year students, indicating that a surgical skills laboratory may be most beneficial for learning repair procedures.

### Limitations

The main limitation of this study is the small sample, and the fact that we did not describe performance results before or after the simulation-based workshop.

### Generalizability

We consider this simulation model as one of the curricular tools for resident education, helping in improving the quality of life of anal sphincter injury patients.

### Suggestions

Further study is needed to evaluate the real performance of residents after a simulation-based workshop when repairing anal sphincter injury of patients.

### Conclusion

This innovative perineal simulator improved the self-confidence of residents in identifying the EAS, suturing the anal mucosa, suturing the IAS, suturing the EAS, and evaluating the sphincter ani tone. All residents were satisfied with the structures of the simulation model.

## Figures and Tables

**Table 1.** Obstetrics and gynecology residents’ demographic characteristics

Characteristic	Value
Age (yr)	31±2.8
Gender	
Male	12 (54.5)
Female	10 (45.5)
Year in residency	
3rd	11 (50.0)
4th	11 (50.0)
Frequency of assistance in anal sphincter injury repair	
<3	17 (77.3)
≥3	5 (22.7)
Learning sources	
Lecture	11 (50.0)
Workshop	3 (13.6)
Book	4 (18.2)
Video	4 (18.2)

Values are presented as mean±standard deviation or number (%).

**Table 2.** Residents’ satisfaction with the silicone-latex anal sphincter simulation model

Variable	Mean score±SD (maximum 10 points)
Similarity to the native perineal tissue	8.0±1.0
Flexibility of the simulator tissue	8.0±2.0
Similarity of the anal mucosa to the native perineal tissue	8.0±1.0
Similarity of the internal sphincter ani to the native perineal tissue	8.18±1.09
Similarity of the external sphincter ani to the native perineal tissue	8.0±2.0
Similarity of the transversus perinei muscle to the native perineal tissue	8.0±2.0
Similarity of the bulbocavernosus muscle to the native perineal tissue	8.0±2.0
Similarity of the vaginal mucosa to the native perineal tissue	8.0±1.0
Similarity of the perineal skin to the native perineal tissue	8.0±1.0

SD, standard deviation.

**Table 3.** Residents’ self-confidence before and after the simulation-based workshop

Confidence	Before the simulation-based workshop	After the simulation-based workshop	P-value
Identification of grade of laceration			1.000
Yes	22 (100.0)	22 (100.0)	
No	0	0	
Identification of anal mucosa			0.125
Yes	18 (81.8)	22 (100.0)	
No	4 (18.2)	0	
Identification of IAS			0.070
Yes	11 (50.0)	17 (77.3)	
No	11 (50.0)	5 (22.7)	
Identification of EAS			0.031^a)^
Yes	15 (68.2)	21 (95.5)	
No	7 (31.8)	1 (4.5)	
Suturing anal mucosa			0.001^a)^
Yes	10 (45.5)	21 (95.5)	
No	12 (54.5)	1 (4.5)	
Suturing IAS			0.001^a)^
Yes	4 (18.2)	17 (77.3)	
No	18 (81.8)	5 (22.7)	
Suturing EAS			<0.001^a)^
Yes	3 (13.6)	19 (86.4)	
No	19 (86.4)	3 (13.6)	
Suturing vaginal mucosa			1.000
Yes	21 (95.5)	22 (100.0)	
No	1 (4.5)	0	
Suturing perineal muscle			0.250
Yes	19 (86.4)	22 (100.0)	
No	3 (13.6)	0	
Suturing perineal skin			1.000
Yes	21 (95.5)	22 (100.0)	
No	1 (4.5)	0	
Evaluation of sphincter ani tone			0.016^a)^
Yes	15 (68.2)	21 (95.5)	
No	7 (31.8)	1 (4.5)	
Evaluation of apex of vaginal mucosa			1.000
Yes	20 (90.9)	21 (95.5)	
No	2 (9.1)	1 (4.5)	
Identification of hymen			1.000
Yes	21 (95.5)	22 (100.0)	
No	1 (4.5)	0	
Instrument handling			1.000
Yes	22 (100.0)	22 (100.0)	
No	0	0	
Selection of an appropriate needle and suture			0.125
Yes	13 (59.1)	18 (81.8)	
No	9 (40.9)	4 (18.2)	

Values are presented as number (%). IAS, internal sphincter ani; EAS, external sphincter ani.

a) By McNemar test.

## Footnotes

**Data availability**
Data files are available from Harvard Dataverse: https://doi.org/10.7910/DVN/FH2IMS Dataset 1. Raw data on residents’ satisfaction for the simulation model.

Dataset 2. Raw data on residents’ self-confidence before and after the simulation workshop.

## Supplementary materials

Data files are available from Harvard Dataverse: https://doi.org/10.7910/DVN/FH2IMS

Supplement 1. Full self-confidence questionnaire distributed to 3rd- and 4th-year obstetrics and gynecology resident at Universitas Airlangga from July to November 2021.

Supplement 2. Video tutorial of 4th-degree perineal laceration for 3rd- and 4th-year obstetrics and gynecology residents at Universitas Airlangga from July to November 2021.

Supplement 3. Audio recording of the abstract.
