# Supplementary material for: Obstetrics and gynecology residents’ satisfaction and self-confidence after an anal sphincter injury simulation-based workshop in Indonesia: a pre- and post-intervention comparison study
Source: J Educ Eval Health Prof. 2022 Feb 14;19:4. doi: 10.3352/jeehp.2022.19.4 (PMC8942784; doi:10.3352/jeehp.2022.19.4)
Supplement: Supplementary file 3 — Supplement 1. Full self-confidence questionnaire distributed to 3rd- and 4th-year obstetrics and gynecology resident at Universitas Airlangga from July to November 2021. [file jeehp-19-04-suppl1.docx]

**Pretest and Posttest Self-Confidence Questionnaire**

| Technique | Confidence^a)^ | Unconfidence^a)^ |
| --- | --- | --- |
| Identification grade of laceration |  |  |
| Identification of anal mucosa |  |  |
| Identification internal anal sphincter |  |  |
| Identification external anal sphincter |  |  |
| Suturing anal mucosa |  |  |
| Suturing internal anal sphincter |  |  |
| Suturing external anal sphincter |  |  |
| Suturing vaginal mucosa |  |  |
| Suturing perineal muscle |  |  |
| Suturing perineal skin |  |  |
| Evaluation sphincter ani tone |  |  |
| Evaluation apex of vaginal mucosa |  |  |
| Identification hymen |  |  |
| Instrumental handling |  |  |
| Selection appropriate needle and suture |  |  |

^a)^Choose confidence/uncofidence.
